# Improvements of the productivity and saccharification efficiency of the cellulolytic β-glucosidase D2-BGL in *Pichia pastoris* via directed evolution

**DOI:** 10.1186/s13068-021-01973-3

**Published:** 2021-05-31

**Authors:** Mu-Rong Kao, Su-May Yu, Tuan-H ua David Ho

**Affiliations:** 1 Molecular and Cell Biology, Taiwan International Graduate Program, Academia Sinica and National Defense Medical Center, Taipei, 115 Taiwan; 2 Institute of Molecular Biology, Academia Sinica, Taipei, 115 Taiwan; 3 Institute of Plant and Microbial Biology, Academia Sinica, Taipei, 115 Taiwan; 4 Department of Plant Pathology, National Chung Hsing University, Taichung, 402 Taiwan; 5 Department of Life Sciences, National Chung Hsing University, Taichung, 402 Taiwan; 6 Biotechnology Center, National Chung Hsing University, Taichung, 402 Taiwan

**Keywords:** β-Glucosidase, GH3, Directed evolution, Lignocellulosic biomass, Saccharification

## Abstract

**Background:**

β-Glucosidases are essential for cellulose hydrolysis by catalyzing the final cellulolytic degradation of cello-oligomers and cellobiose to glucose. D2-BGL is a fungal glycoside hydrolase family 3 (GH3) β-glucosidase isolated from *Chaetomella raphigera* with high substrate affinity, and is an efficient β-glucosidase supplement to *Trichoderma reesei* cellulase mixtures for the saccharification of lignocellulosic biomass.

**Results:**

We have carried out error-prone PCR to further increase catalytic efficiency of wild-type (WT) D2-BGL. Three mutants, each with substitution of two amino acids on D2-BGL, exhibited increased activity in a preliminary mutant screening in *Saccharomyces cerevisiae*. Effects of single amino acid replacements on catalysis efficiency and enzyme production have been investigated by subsequent expression in *Pichia pastoris*. Substitution F256M resulted in enhancing the tolerance to substrate inhibition and specific activity, and substitution D224G resulted in increasing the production of recombinant enzyme. The best D2-BGL mutant generated, Mut M, was constructed by combining beneficial mutations D224G, F256M and Y260D. Expression of Mut M in *Pichia pastoris* resulted in 2.7-fold higher production of recombinant protein, higher *V*_max_ and greater substrate inhibition tolerance towards cellobiose relative to wild-type enzyme. Surprisingly, Mut M overexpression induced the ER unfolded protein response to a level lower than that with WT D2 overexpression in *P. pastoris*. When combined with the *T. reesei* cellulase preparation Celluclast 1.5L, Mut M hydrolyzed acid-pretreated sugarcane bagasse more efficiently than WT D2.

**Conclusions:**

D2-BGL mutant Mut M was generated successfully by following directed evolution approach. Mut M carries three mutations that are not reported in other directed evolution studies of GH3 β-glucosidases, and this mutant exhibited greater tolerance to substrate inhibition and higher *V*_max_ than wild-type enzyme. Besides the enhanced specific activity, Mut M also exhibited a higher protein titer than WT D2 when it was overexpressed in *P. pastoris*. Our study demonstrates that both catalytic efficiency and productivity of a cellulolytic enzyme can be enhanced via protein engineering.

**Supplementary Information:**

The online version contains supplementary material available at 10.1186/s13068-021-01973-3.

## Background

Lignocellulosic biomass is considered a sustainable source of value-added products such as biofuels. Physical and chemical pre-treatments are required to break down lignocellulose into lignin, hemicellulose and cellulose [1]. Cellulose fibers are hydrolyzed by cellulolytic enzymes to release the glucose that can be transformed into biofuels and other chemicals via catalytic conversion [2]. Synergism among three types of cellulase is required to complete the process of biological hydrolysis. Endo-glucanases and exo-glucanases degrade cellulose into cello-oligomers and cellobiose, whereas β-glucosidases play a crucial role in releasing the final product (i.e., glucose) from cellobiose [3].

β-Glucosidases can be categorized based on a substrate preference for aryl-β-D-glucosides (including *p*NPG), alkyl-β-D-glucosides (including cellobiose), or those with broader preference [4]. Based on CAZyme classification (Carbohydrate Active enZymes database [5]), the majority of β-glucosidases belong to glycoside hydrolase family 1 (GH1) with a classical (α/β)_8_ TIM-barrel structure [6] or to glycoside hydrolase family 3 (GH3) enzymes having one to four unique structural domains [7–10]. Among microbial β-glucosidases, the fungal GH3 enzymes have been widely studied for their high catalytic efficiency towards cellobiose and cello-oligomers, and they are considered to be the most suitable β-glucosidase supplement for cellulase mixtures used in biomass saccharification [11–13].

To make the biocatalytic process economically feasible, efforts are on-going to discover novel β-glucosidases with enhanced performance. Protein engineering can further improve the biochemical properties of these enzymes. Structure- and sequence-based methods have been applied successfully to generate mutant cellulases exhibiting enhanced catalytic efficiency and thermostability [14–16]. Mutants with increased cellulolytic activity have indeed been discovered among *Aspergillus* GH3 β-glucosidases [17, 18]. However, application of these mutations discovered in *Aspergillus sp.* may not provide the same beneficial effects to other structurally and phylogenetically distant GH3 enzymes. Therefore, random mutagenesis coupled with an efficient high-throughput screening process represents a powerful molecular tool for discovering new beneficial mutations.

We previously identified the GH3 β-glucosidase D2-BGL from the fungus *Chaetomella raphigera* based on its dynamic synergism with *T. reesei* cellulases to degrade cellulose efficiently [19]. Functional and structural analyses have since revealed that *P. pastoris*-expressed D2-BGL exhibits high substrate affinity, and its specific glycosylation patterns are required for enzyme production [20]. The study further showed that use of D2-BGL as a β-glucosidase supplement in a *T. reesei* cellulase mixture shortened the time necessary for bioethanol production via a semi-simultaneous saccharification and fermentation process. Here, we improved D2-BGL catalytic efficiency by adopting a random mutagenesis approach. We generated D2-BGL mutants carrying beneficial mutations identified from transformants displaying improved enzymatic activity, and then evaluated their efficiency as a β-glucosidase supplement in the saccharification of acid-pretreated biomass.

## Results and discussion

### Expression of recombinant D2-BGL in *S. cerevisiae* and *P. pastoris*

*Saccharomyces cerevisiae* is commonly used for the construction of mutant libraries via random mutagenesis because of its ability to reconstitute linearized DNA fragments into plasmids by homologous recombination [21]. *P. pastoris* is frequently used in industry for large-scale production of recombinant enzyme [22]. Thus, we intended to express β-glucosidase D2-BGL in both expression systems, and examine their characteristics. First, we tested the suitability of *S. cerevisiae* to express wild-type D2-BGL (WT D2). SDS-PAGE analysis was performed with purified *S. cerevisiae*-expressed WT D2 (Sc D2-BGL) and *P. pastoris*-expressed WT D2-BGL (Pp D2-BGL) (Additional File 1: FigureS1). Both Pp D2-BGL and Sc D2-BGL presented a smeared protein profile in SDS-PAGE, but the Sc D2-BGL profile showed a higher molecular weight (proteins between 100 and > 180 kDa) than that of Pp D2-BGL. However, upon deglycosylation via endoglycosidase (Endo H) treatment, the profiles of both Pp D2-BGL and Sc D2-BGL manifested as single bands of identical molecular weight of about 76 kDa, indicating that the degree of hyper-mannosylation in Sc D2-BGL is higher than that in Pp D2-BGL. Sc D2-BGL presented lower enzyme activity than Pp D2-BGL (0.3 ± 0.02 U/mL vs 2.33 ± 0.57 U/mL) in 2-day culture supernatants. *S. cerevisiae*-produced D2-BGL also had lower specific activity than that produced by *P. pastoris* (144.6 ± 20.2 U/mg vs 214.3 ± 25.0 U/mg at 10 mM cellobiose), suggesting that hyper-glycosylation probably affects negatively the enzyme activity in Sc D2-BGL. The protein production level is estimated to be 2 mg/L for Sc D2-BGL and 11 mg/L for Pp D2-BGL. Despite the low recombinant protein production observed for Sc D2-BGL, its enzyme activity was still detectable in the supernatant. As we expected, *S. cerevisiae* was deemed to be a suitable expression host for constructing the D2-BGL mutant library, whereas *P. pastoris* was deemed to be a better producer of recombinant enzyme in large-scale. Glycosylation in the two organisms may affect enzyme activity, so the catalytic efficiency of *S. cerevisiae*-expressed D2-BGL mutants must be confirmed later in *P. pastoris*.

### Characterization of D2-BGL mutant F256M

**Fig. 1:**
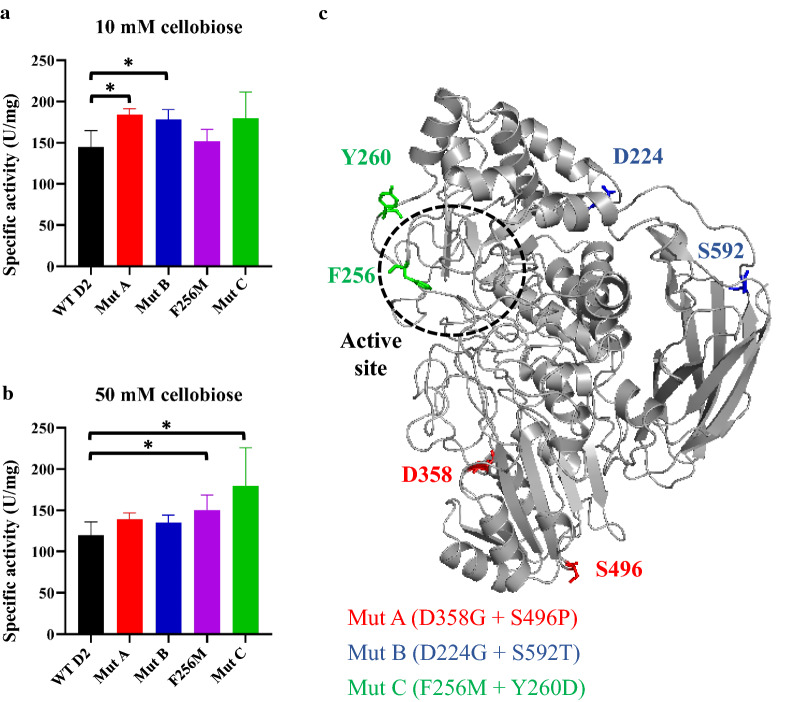
*S. cerevisiae-*expressed D2-BGL mutants show higher specific activity than the wild-type D2-BGL (WT D2). **a**, **b** Specific activity was determined at 10 mM cellobiose (**a**) and 50 mM cellobiose (**b**) with Mut A (D358G + S496P, in red), Mut B (D224G + S592T, in blue) and Mut C (F256M + Y260D, in green). **c** Locations of the six amino acid replacements on the three-dimensional protein structure of D2-BGL (PDB: 6JXG). Experiments were performed in triplicate, and error bars represent the standard deviation. *: *P* value < 0.05 (*t* test)

Among GH3 β-glucosidases, the substrate-binding subsite + 1 is formed by a triad of hydrophobic amino acid residues, with W34, F256 and Y444 being involved in the binding of the non-reducing sugar moiety of cellobiose in D2-BGL [20]. Substitution of residue Y305 in *S. cerevisiae*-expressed *Aspergillus niger* β-glucosidase BGL1 with cysteine (C) or glycine (G) increased both apparent *K*_m_ (_app_ *K*_m_) and apparent *V*_max_ (_app_*V*_max_) towards cellobiose and maintained the reaction rate at high cellobiose concentrations [17]. Since the substrate-binding residue F256 in D2-BGL is equivalent to Y305 in *A. niger* BGL1, we generated the F256C mutant in *S. cerevisiae* to see whether the replacement of F256 by a cysteine (C) with a short side-chain may increase enzyme activity. However, the F256C mutant presented lower specific activity than WT D2 (112 ± 6 vs 144.6 ± 20.2 U/mg at 10 mM cellobiose). Next, we substituted F256 with tyrosine (Y) or methionine (M), both of which are hydrophobic and have a similar side-chain length to phenylalanine (Additional File 1: FigureS2). We found that specific activity was dramatically reduced in the *S. cerevisiae*-expressed F256Y mutant (83.8 ± 4.8 vs 144.6 ± 20.2 U/mg in WT D2 at 10 mM cellobiose). The specific activity of the F256M mutant at 10 mM cellobiose was not significantly different to WT D2 (151.5 ± 14.9 vs 144.6 ± 20.2 U/mg, Fig. 1a), but exhibited 26% higher specific activity relative to WT D2 at 50 mM cellobiose (150.6 ± 18 vs 119.6 ± 16.4 U/mg, Fig. 1b), indicating that substituting phenylalanine with methionine at residue 256 enhances hydrolytic efficiency at high cellobiose concentrations. As the position of F256 depends on residue W34 via hydrophobic interactions between their aromatic rings, we suggest that the aliphatic side-chain in methionine confers greater structural flexibility than phenylalanine when facing W34 and cellobiose, resulting in a reduced effect of substrate inhibition on the F256M mutant.

### Screening of D2-BGL mutants with improved cellobiose hydrolysis efficiency

We performed error-prone PCR (ePCR) on WT D2 and the F256M mutant as templates to generate two mutant libraries. The enzyme activity of culture supernatant for each transformant was evaluated by the measurement of OD_405_ value with *p*NPG assay. In the first library, 12,411 of 26,912 transformants (46%) generated with ePCR products from WT D2 presented a OD_405_ lower than AVE_WT_ − 2 × STDEV_WT_ in 96-well plate culture. Among 440 transformants (2%) that presented a OD_405_ higher than AVE_WT_ + 2 × STDEV_WT_, 18 transformants with the highest OD_405_ value were selected for flask culturing and two of them (Mut A and Mut B) still showed enhanced cellobiase activity. Mut A (D358G + S496P) and Mut B (D224G + S592T) showed 24% and 23% higher specific activity than WT D2 at 10 mM cellobiose, respectively (Fig. 1a). Mut A harbors three mutations in D2-BGL sequence (2166 bp) resulting in the substitution of two amino acid residues (D358G and S496P) in the α/β sandwich domain of D2-BGL, whereas Mut B has five mutations resulting in one substitution (D224G) in the TIM-barrel-like domain and one (S592T) in the fibronectin type III-like domain (Fig. 1c and Additional File 1: TableS1). The four amino acid substitutions observed in the Mut A and Mut B mutants are localized on the protein surface.

To obtain D2-BGL mutants with both enhanced enzyme activity and tolerance to substrate inhibition, we generated the second library with ePCR products from the F256M mutant that exhibited higher specific activity than WT D2 at high cellobiose concentration. Only 4 of 1848 transformants (0.2%) showed enhanced enzyme activity in 96-well plate culture, and only one transformant (Mut C) still showed enhanced cellobiase activity in flask culture. Mut C (F256M + Y260D) showed similar specific activity with WT D2 at 10 mM cellobiose but higher specific activity (up to 50%) at 50 mM cellobiose (Fig. 1a, b). Mut C has two mutations resulting in an additional amino acid replacement (Y260D) in the same loop that harbors residue F256 (Fig. 1c and Additional File 1: TableS1). We hypothesize that the Y260D + F256M double mutation modifies the flexibility of the short loop from residues 258 to 264, thereby influencing the accessibility of the active site to cellobiose.

**Fig. 2:**
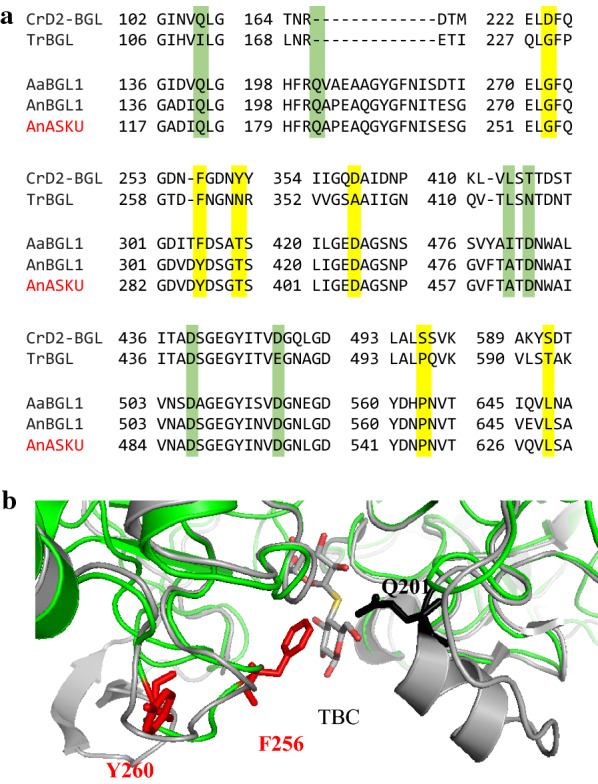
Sequence and structure analyses of D2-BGL and *Aspergillus* GH3 β-glucosidases. **a** Alignment between D2-BGL (CrD2-BGL), *Trichoderma reesei* BGL (TrBGL), *A. aculeatus* BGL1 (AaBGL1), *A. niger* BGL1 (AnBGL1) and *A. niger* ASKU28 BGL (AnASKU). Mutation points discovered in D2-BGL and *Aspergillus* enzymes are highlighted in yellow or green, respectively. **b** Structure superposition between D2-BGL (PDB: 6JXG, in green) and AaBGL1 (PDB: 4IIH, in grey) at the active site. F256 and Y260 in D2-BGL are presented in red stick, and Q201 in AaBGL1 is presented in black stick. TBC: thiocellobiose

It was previously shown that substitution of acidic amino acid residues D463, D487 and D496 by alanine (A) in *A. niger* ASKU28 β-glucosidase enhanced the catalytic efficiency (i.e., *k*_cat_/*K*_m_) up to 2.3-fold higher than wild-type enzyme [23]. A quadruple mutant of *A. niger* β-glucosidase BGL1, including V22D mutation of the α-mating factor secretion signal peptide and Y305C mutation of the substrate-binding subsite + 1, had an _app_*V*_max_ value threefold greater than that of wild-type enzyme [17]. Moreover, an *A. aculeatus* β-glucosidase AaBGL1 mutant Q201E, in which the mutation is near the active site, exhibited a catalytic efficiency towards cellobiose that was 2.7-fold greater than wild-type [18]. Here, we have identified six amino acids whose replacement resulted in enhanced enzyme activity. Sequence alignment analysis revealed that only one of these six residues (i.e., D358) is a conserved amino acid in D2-BGL and *Aspergillus* β-glucosidases (Fig. 2a). Amino acid S496 is replaced in Mut A by a proline (P), a conserved residue at the relative position in TrBGL (P496), AaBGL1 (P563), AnBGL1 (P563) and AnASKU (P544) in the α/β sandwich domain. Similarly, the D224 in Mut B is replaced by a glycine (G), and this glycine is a conserved residue for *T. reesei* and *Aspergillus* β-glucosidases (i.e., TrBGL G229, AaBGL1 G272, AnBGL1 G272 and AnASKU G253) in the TIM barrel-like domain.

In our previous study, we have proposed to classify fungal GH3 β-glucosidases into two clades based on their protein lengths [20]. D2-BGL and *T. reesei* BGL belong to the clade II enzymes with fewer than 800 amino acid residues, whereas *Aspergillus* enzymes belong to clade I with higher residue number. Clade I and II enzymes show structure diversity at the entry region of the active site. The loop from residues 166 to 168 in D2-BGL is shorter than the same loop from residues 200 to 215 in *A. aculeatus* AaBGL1 (Fig. 2a). Consequently, there is no homolog of AaBGL1 Q201 at the same position in the structure of D2-BGL (Fig. 2b). The F256 and Y260 in D2-BGL are on a loop that is one amino acid shorter than the corresponding loop in clade I enzymes, and this short loop is crucial for the high substrate affinity in D2-BGL [20]. Since clade I and II enzymes share low similarity of protein sequence and structure in the entry region of the active site, we expect that our discovery would provide new possibilities to improve clade II GH3 β-glucosidases via a structure-based mutagenesis approach.

### Comparisons among *P. pastoris*-expressed D2-BGL mutants

In our previous study, we demonstrated that *P. pastoris*-expressed D2-BGL is an efficient cellulolytic β-glucosidase with a high substrate affinity for cellobiose, and its production can be upscaled using a 1-ton bioreactor [20]. Industrial-level applications of D2-BGL in the saccharification of lignocellulosic biomass could become even more economically profitable if its enzyme activity and/or production could be further improved. For this purpose, we generated nine mutants—including Mut A, Mut B, Mut C and their six single amino acid replacement mutants—and transformed them into *P. pastoris*. Mut C (F256M + Y260D) and the F256M mutant exhibited increased enzyme activity in the culture supernatant (i.e., up to 77% higher than WT D2 in Mut C) (Table 1). Mut C and the F256M mutant also presented higher specific activity than WT D2 (253 ± 29 and 301 ± 26 vs 213 ± 8 U/mg in WT D2), which suggests that amino acid replacements in the active site region may enhance the enzyme activity in D2-BGL mutants.

**Table 1:** Enzyme activity in culture supernatant and specific activity for *P. pastoris*-expressed wild-type and mutant D2-BGL

*Pichia pastoris *strain	Mutation	Activity^a, b^ (U/mL)	Relative activity^c^ (%)	Specific activity^b^ (U/mg)
WT D2		2.31 ± 0.20	100 ± 9	213 ± 8
Mut A	D358G + S496P	2.24 ± 0.04	97 ± 2	218 ± 1
D358G	D358G	1.77 ± 0.21	77 ± 9	164 ± 10
S496P	S496P	1.94 ± 0.12	84 ± 5	182 ± 8
Mut B	D224G + S592T	4.29 ± 0.42	186 ± 18	229 ± 19
D224G	D224G	4.08 ± 0.36	177 ± 16	202 ± 19
S592T	S592T	1.72 ± 0.51	75 ± 22	195 ± 13
Mut C	F256M + Y260D	4.09 ± 0.52	177 ± 23	253 ± 29
F256M	F256M	3.01 ± 0.59	130 ± 25	301 ± 26
Y260D	Y260D	1.92 ± 0.38	83 ± 17	189 ± 11
D224G F256M	D224G + F256M	5.70 ± 0.26	247 ± 11	236 ± 14
D224G Y260D	D224G + Y260D	4.63 ± 0.70	200 ± 30	192 ± 4
Mut M	D224G + F256M + Y260D	7.5 ± 0.75	325 ± 33	261 ± 23

^a^Enzyme activity is measured with a 3-day culture supernatant

^b^Enzyme activity and specific activity are determined at 10 mM cellobiose

^c^Relative activity is determined based on the enzyme activity of WT D2

Mut B and the D224G mutant exhibited increased activity in the culture supernatant up to 86% higher than WT D2 (Table 1). However, the specific activity is similar between these two mutants and WT D2. Since the production of recombinant enzyme is estimated to be 18.7 mg/L in Mut B and 20.2 mg/L in the D224G mutant (vs 10.8 mg/L in WT D2), respectively, we hypothesized that the substitution D224G may enhance the expression of recombinant D2-BGL. To confirm this hypothesis, we generated double mutants (D224G + F256M) and (D224G + Y260D). Inclusion of the D224G mutation also increased the enzyme activity of these two double mutants without improving their specific activities in comparison with the mutants with single mutation F256M or Y260D (Table1). The production of recombinant enzyme is estimated to be 24.2 mg/L in the (D224G + F256M) mutant and 24.1 mg/L in the (D224G + Y260D) mutant, which is more than twofold higher than that in the F256M mutant (10 mg/L) and the Y260D mutant (10.2 mg/L), respectively. These results suggest that the mutation D224G could increase the enzyme expression for different D2-BGL mutants in *P. pastoris*.

### Production of recombinant Mut M D2-BGL

**Fig. 3:**
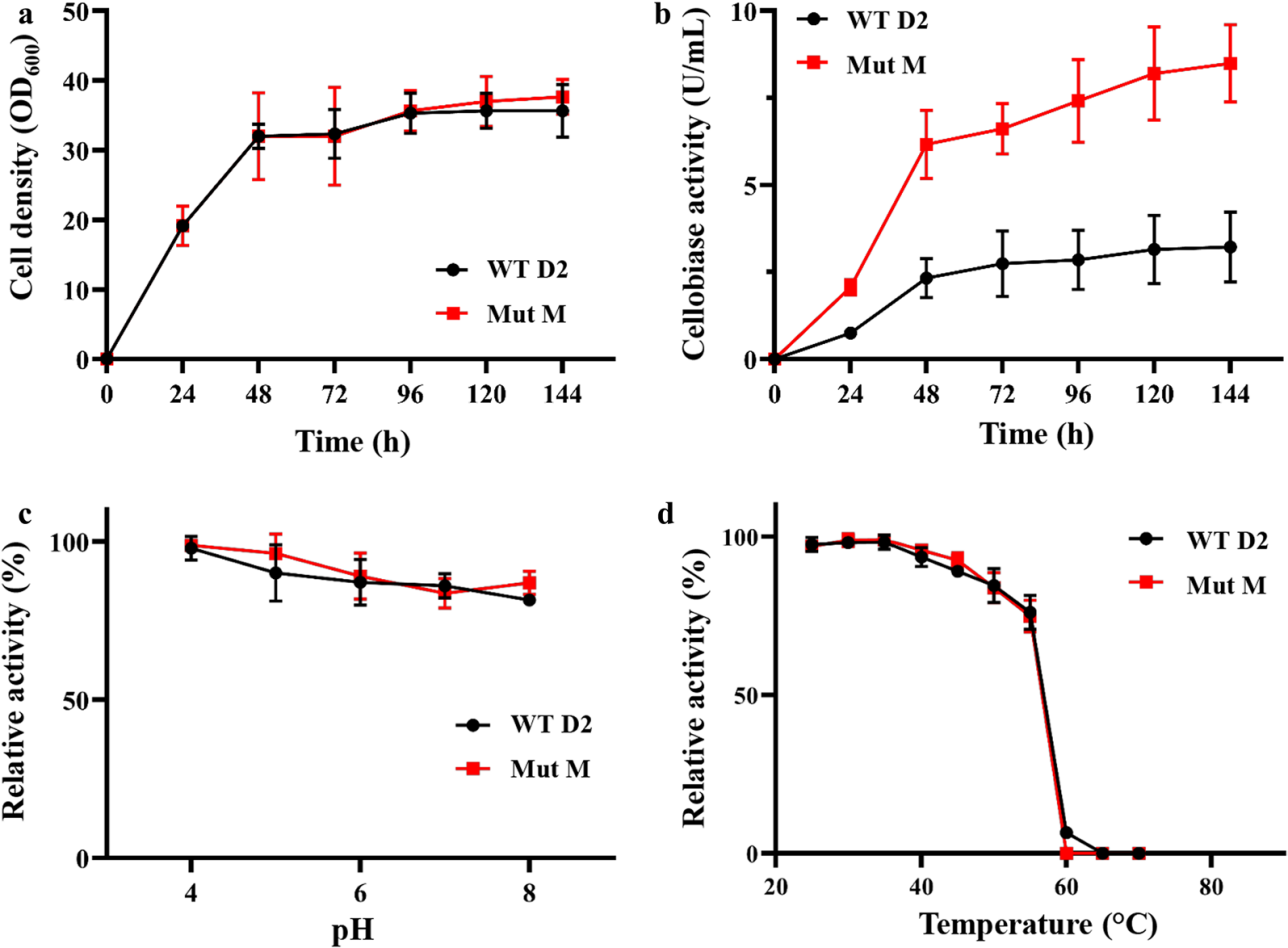
Mut M expressed in *P. pastoris* shows higher cellobiase activity than that of WT. **a** Growth curves of *P. pastoris* strains expressing WT D2 and Mut M. **b** Cellobiase activity measured with culture supernatant from WT D2 and Mut M at 10 mM cellobiose. **c** Stability assay with samples incubated at different pH with preservation at 4 °C for 24 h. **d** Stability assay with samples incubated at different temperatures for 4 h. Experiments were performed at least in triplicate, and error bars represent the standard deviation

To verify if the beneficial effect of the enhancement of activity is cumulative with the increase of enzyme expression, next we generated the Mut M mutant with triple mutation (F256M + Y260D + D224G). In flask culture, cell densities across a 6-day incubation period were similar between *P. pastoris* strains expressing WT D2 and Mut M (Fig. 3a). However, the cellobiase activity measured in the culture supernatant of Mut M was significantly higher than that of WT D2 after 24-h of cultivation (Fig. 3b). The pH stability and thermostability of WT D2 and Mut M are similar (Fig. 3c, d), indicating that the increased enzyme activity of mutant enzyme was not due to enhanced protein stability.

**Fig. 4:**
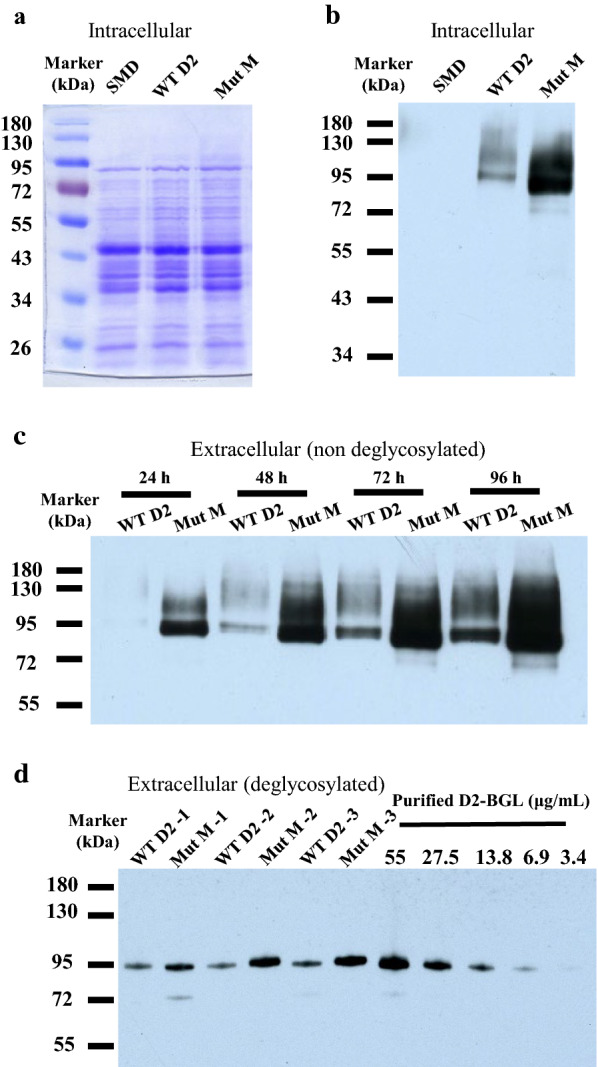
The protein level of Mut M is higher both in cellulo and in culture medium. **a** SDS-PAGE analysis of total intracellular proteins extracted from *P. pastoris* wild-type SMD 1168 (SMD), WT D2 and Mut M strains. Ten micrograms of proteins were loaded for each sample. **b** Western blotting analysis of total intracellular proteins with specific antibodies anti-D2-BGL. **c** Western blotting of culture supernatant collected from WT D2 and Mut M at 24-h interval. Twenty microliters of supernatant were loaded in each well. **d** Western blotting with deglycosylated culture supernatant from WT D2 and Mut M. Culture supernatants from three different clones of Mut M (Mut M -1, -2 and -3) and WT D2 (WT D2 -1, -2 and -3) were used. Four microliters of deglycosylated sample were loaded in each well. Samples were collected from a 3-day culture. Protein concentration was estimated using purified D2-BGL as standard

During the flask culture, we found that Mut M presented higher enzyme activity (7.5 ± 0.75 vs 2.31 ± 0.2 in WT D2) and specific activity (261 ± 23 vs 213 ± 8 in WT D2), indicating that the production of Mut M is 2.7-fold higher in *P. pastoris* (28.8 vs 10.8 mg/L in WT D2) (Table 1). In addition, Mut M exhibited a specific activity similar to those of Mut C and the F256M mutant. To confirm that Mut M has enhanced enzyme expression, we applied a Western blotting-based technique to evaluate the production of recombinant enzyme at intracellular and extracellular levels in *P. pastoris* strains expressing WT D2 or Mut M. The SDS-PAGE gel stained with Coomassie Brilliant Blue revealed similar profiles for total intracellular proteins extracted from *P. pastoris* wild-type strain SMD 1168 (SMD) and strains expressing D2-BGL WT D2 or Mut M (Fig. 4a), but protein amounts of recombinant D2-BGL visualized using Western blotting analysis were clearly higher for Mut M than WT D2 (Fig. 4b). Our time-course Western blotting analysis also revealed that D2-BGL production increased from the first day of cultivation for both the WT D2 and Mut M strains, but protein production was always higher for Mut M than WT D2 in the culture supernatant (Fig. 4c). To better evaluate the D2-BGL concentration, we performed the Western blotting with the deglycosylated culture supernatants from WT D2 and Mut M, and we used a serial dilution of purified and deglycosylated WT D2 to establish a calibration curve (Fig. 4d). The blot image revealed that all samples exhibited single bands of 95 kDa, which indicates that the deglycosylation was not complete but sufficient to eliminate the smearing caused by hyper-mannosylation. After 72 h, production of D2-BGL was estimated to be 2.4-fold higher for the Mut M compared to the WT D2 strain (31 ± 7 vs 13 ± 2 mg/L), which is close to the value calculated from activity assays (i.e., 2.7-fold).

Variation of recombinant protein expression among transformants is a phenomenon commonly observed in *P. pastoris*. This “clonal variability” is caused by unspecific transgene insertion into the genomic DNA, which could consequently affect the physiology of the yeast as well as the protein secretion levels [24, 25]. To ensure the comparison of enzyme expression between WT D2 and Mut M is not biased by clonal variability, WT D2 and Mut M clones were selected from three independent transformations. Growth and enzyme activity curves determined with those clones were in the range of variations represented in Fig. 3a, b. We have also verified that the transgene was correctly inserted into the glyceraldehyde-3-phosphate dehydrogenase (GAP) promoter region on the chromosome 2 of *P. pastoris* using PCR analysis (data not shown).

**Fig. 5:**
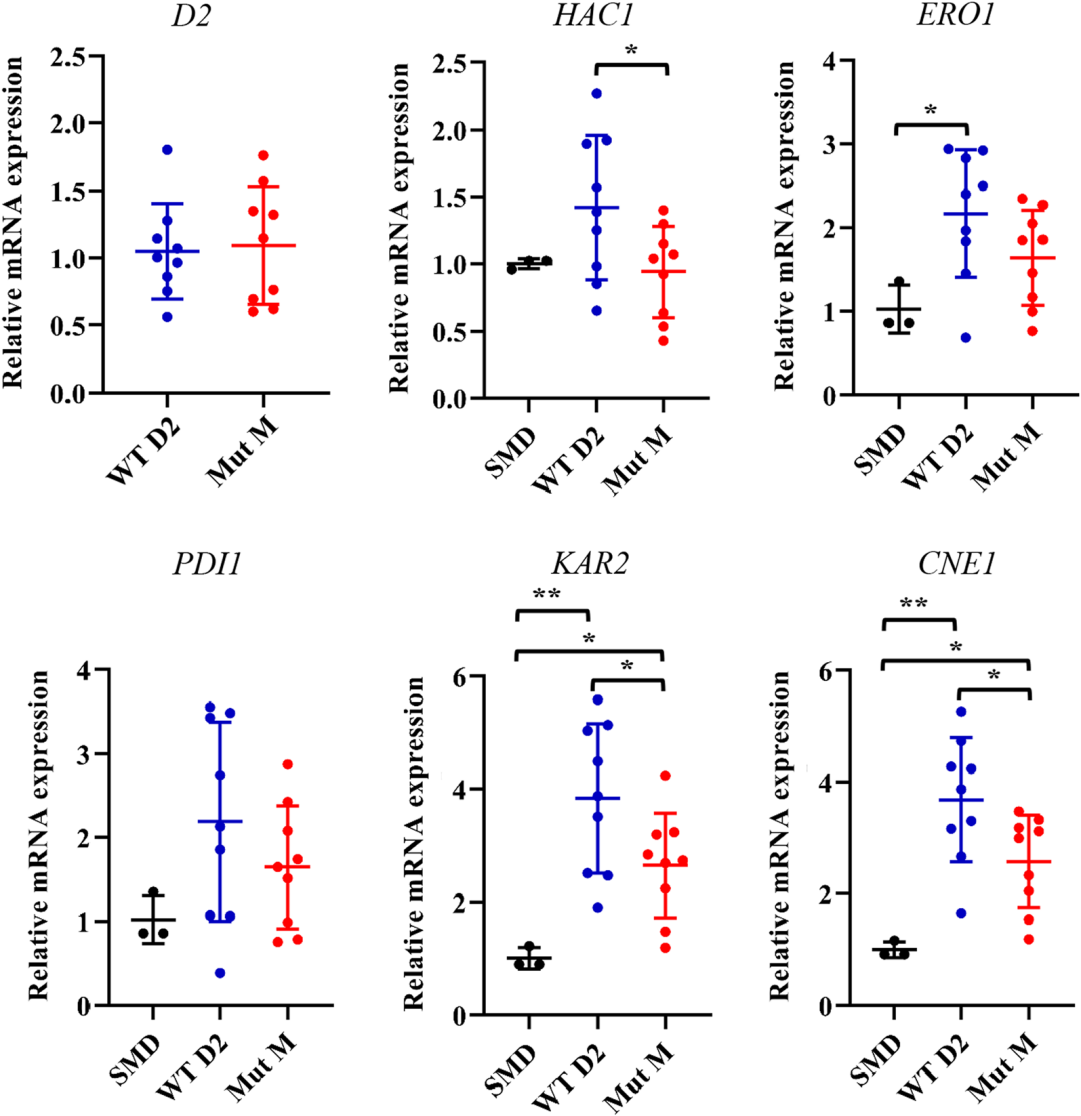
Production of Mut M mutant enzyme induces a lower unfolded protein response in *P. pastoris*. Relative expression of D2-BGL (*D2*) and genes related to UPR and ER quality control were determined using qPCR. *HAC1*: UPR activating transcription factor; *KAR2*: ER chaperone; *PDI1*: protein disulfide isomerase; *ERO1*: Pdi oxidase; *CNE1*: calnexin (ER chaperone). Experiments were performed at least in triplicate, and error bars represent the standard deviation. Relative expression was determined using the 2^−ΔΔCT^ method with expression level of ACT1 in SMD 1168 strain as reference. *: *P *value < 0.05; **: *P* value < 0.01 (*t* test)

In eukaryotic cells, overexpression of recombinant proteins leads to increased accumulation of unfolded proteins in endoplasmic reticulum (ER), which induces ER stress [26]. To maintain ER homeostasis, the unfolded protein response (UPR) is activated to up-regulate the protein folding or degradation processes. In *P. pastoris*, over-expression of recombinant anti-CD3 immunotoxin was reported to enhance the intracellular level of Kar2p, a chaperone involved in UPR [27]. Heterologous expression of an antibody Fab fragment upregulated the gene expression level of UPR-related proteins such as Kar2p, protein disulfide isomerase (Pdi), ER oxidoreduction 1 Pdi oxidase (Ero1) and calnexin (Cne1) [28]. As the production of Mut M is increased at both extracellular and intracellular levels, we would like to know if the level of UPR was higher in Mut M-expressing *P. pastoris* strain compared to the WT D2-expressing strain. For this purpose, we conducted the qPCR analysis to determine the relative expression of genes encoding Kar2p (*KAR2*), Pdi (*PDI1*), Ero1 (*ERO1*) and Cne1 (*CNE1*) as well as the D2-BGL (*D2*) and the transcription factor Hac1p (*HAC1*). Relative expression of *D2* was similar between WT D2 and Mut M strains (Fig. 5), implying that the Mut M mutations have no effect on transcription of *D2* or post-transcriptional processing of its transcripts. Heterologous expression of WT D2 and Mut M enzymes in *P. pastoris* showed a trend of upregulated UPR-related genes. Compared to untransformed *P. pastoris* SMD1168 strain (SMD), there was no change in transcript levels of *HAC1* or *PDI1* upon heterologous expression of WT D2. However, constitutive expression of WT D2 significantly increased the expression levels of *ERO1*, *KAR2* and *CNE1*. Surprisingly, relative expression of UPR-related genes was lower for over-expression of the Mut M variant than for WT D2, despite the Mut M mutations induced high-level production of recombinant enzyme (Figs. 4, 5). Accordingly, relative expression of *CNE1* was significantly lower for the strain overexpressing Mut M compared to WT D2.

Based on these results, we speculate that the Mut M mutations could facilitate protein folding and sorting in ER, resulting in a lower level of UPR, as indicated by the reduced expression of *KAR2* and *CNE1* in the Mut M strain relative to WT D2. Moreover, as intracellular D2-BGL protein amounts were higher in the Mut M strain than in WT D2 (Fig. 4b), we suggest that the time to traffic recombinant D2-BGL from ER to Golgi is shorter in the Mut M strain. Alternatively, the diminished UPR could lead to less protein degradation in ER and consequently higher intracellular levels of recombinant protein.

*Pichia pastoris* is widely used for large-scale expression of industrial enzymes. Numerous experimental strategies have been adopted to improve recombinant protein yields, including optimization of codon usage, selection of suitable promoters, and enhancements of gene copy number [29]. As ER-resident chaperones participate actively in protein folding and secretion, recent research efforts have focused on improving recombinant protein production via over-expression of *HAC1*, *KAR2*, *PDI1* or factors involved in protein secretion in *P. pastoris* [30–33]. Here, we report that production of recombinant Mut M D2-BGL is 2.7-fold higher than WT D2 in *P. pastoris* and does not trigger a high level of UPR as WT D2-BGL does. We suggest that protein engineering represents an effective approach to improving the stability and folding of nascent protein in ER, resulting in an increase of recombinant protein production in *P. pastoris*.

### Kinetic properties of Mut M are also improved

We determined kinetic parameters for *P. pastoris*-expressed WT D2, as well as F256M and Mut M mutant proteins, to evaluate their catalytic efficiency towards cellobiose and *p*NPG (Table 2 and Additional File 1: FigureS3). Values of *V*_max_, *K*_m_ and *K*_i substrate_ towards cellobiose were increased in F256M (584 ± 13 U/L, 1.51 ± 0.13 mM and 208 ± 28 mM) relative to WT D2 (Table 2), revealing that the single amino acid replacement of the substrate-binding residue F256 by methionine (M) enhances both enzymatic activity and tolerance to substrate inhibition, but reduces substrate affinity. Compared to WT D2, Mut M showed higher *V*_max_ and *K*_m_ values towards cellobiose (469 ± 13 U/L and 1.69 ± 0.17 mM vs 371 ± 15 U/L and 1.28 ± 0.2 mM) but lower *V*_max_ and *K*_m_ towards *p*NPG (373 ± 30 U/L and 0.18 ± 0.05 mM vs 575 ± 110 U/L and 0.31 ± 0.14 mM), implying that Mut M exhibits a preference for natural over artificial substrate. Moreover, we found that Mut M was more sensitive to glucose inhibition, resulting in a lower *K*_i glucose_ value than for WT D2 (1.59 ± 0.07 vs 3.23 ± 1.08 mM). However, the *K*_i substrate_ values were higher for Mut M than for WT D2 (200 ± 32 vs 133 ± 25 mM for cellobiose and 3.21 ± 0.56 vs 2.19 ± 0.75 mM for *p*NPG), indicating that the mutant protein has higher enzymatic activity at high substrate concentrations due to its greater tolerance to substrate inhibition than WT D2. The higher sensitivity of Mut M D2-BGL to the reaction product glucose may not present a practical issue if the glucose is quickly utilized in the subsequent fermentation process.

**Table 2:** Kinetic parameters of WT D2, F256M and Mut M enzymes

	WT D2	F256M	Mut M
Substrate			
Cellobiose			
* V*_max_ (U/L)	371 ± 15	584 ± 13	469 ± 13
* K*_m_ (mM)	1.28 ± 0.20	1.51 ± 0.13	1.69 ± 0.17
* K*_*i* substrate_ (mM)	133 ± 25	208 ± 28	200 ± 32
* p*NPG			
* V*_max_ (U/L)	575 ± 110	ND	374 ± 30
* K*_m_ (mM)	0.31 ± 0.14	ND	0.18 ± 0.05
* K*_*i* substrate_ (mM)	2.19 ± 0.75	ND	3.21 ± 0.56
* K*_*i* glucose_ (mM)	3.23 ± 1.08	ND	1.59 ± 0.07

*ND* not determined

### Saccharification of acid-pretreated biomass with Mut M

**Fig. 6:**
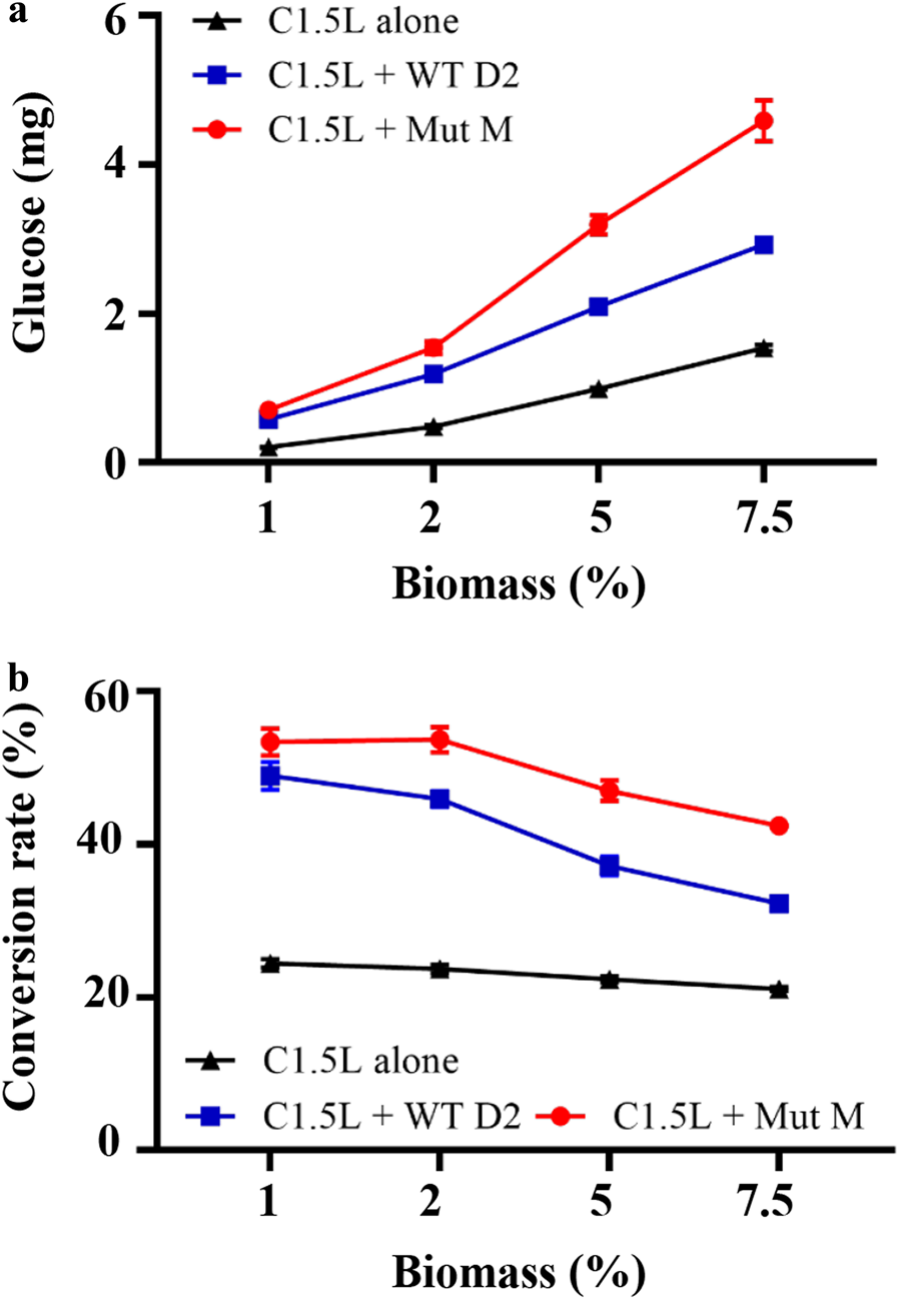
Mut M exhibits better saccharification efficiency than WT D2 at high biomass concentrations. **a** The glucose released after a 6-h hydrolysis process. Sugarcane bagasse saccharification was carried out with 0.06 FPU C1.5L supplemented with 0.3 μg WT D2 or Mut M per 1% of biomass. **b** Final cellulose-to-glucose conversion rate calculated at the end of 72-h hydrolysis process. Experiments were performed in triplicate, and error bars represent the standard deviation

In our kinetics study, Mut M exhibited enhanced specific activity at high cellobiose concentrations compared to WT D2 (37% increase at 50 mM cellobiose vs 23% increase at 10 mM cellobiose). The mutant protein also showed higher hydrolysis efficiency towards 1 mM cellotriose (262.5 ± 11.6 vs 224.2 ± 2 U/mg in WT D2). To evaluate the performance of Mut M in the saccharification of lignocellulosic biomass, we prepared cellulase mixtures with Celluclast 1.5L (C1.5L, used as source of endo-glucanases and exo-glucanases) supplemented with WT D2 or Mut M. We assayed the saccharification of sugarcane bagasse with an equal protein amount of WT D2 and Mut M. The mixture supplemented with D2-BGL exhibited better saccharification efficiency than that with C1.5L alone (Fig. 6a). The glucose obtained from sugarcane bagasse hydrolysis with Mut M was 22% higher than that for WT D2 (0.70 ± 0.04 vs 0.58 ± 0.02 mg) for 1% biomass, and 57% higher (4.59 ± 0.27 vs 2.92 ± 0.08 mg) for 7.5% biomass after a 6-h hydrolysis process. The conversion rate after a 72-h hydrolysis process decreased when the biomass concentration increased (Fig. 6b). However, the conversion rate obtained with C1.5L + Mut M decreased slower than the one with C1.5L + WT D2 (53 to 42% vs 49 to 32%). These results indicate that Mut M enzyme has better saccharification efficiency than WT D2-BGL at high biomass concentrations and that use of Mut M as a β-glucosidase supplement could more efficiently enhance the saccharification of acid-pretreated sugarcane bagasse.

## Conclusions

We have demonstrated that directed evolution successfully generated a triple mutant, Mut M, of the β-glucosidase D2-BGL, which exhibits the highest increase of enzymatic activity in the culture supernatant relative to wild-type and mutant enzymes expressed in *P. pastoris.* Mut M displays higher *V*_max_ and *K*_i substrate_ values, resulting in a catalytic efficiency greater than that of wild-type enzyme. Production of recombinant Mut M is increased 2.7-fold over wild-type enzyme, probably due to a diminished unfolded protein response in *P. pastoris*. When used as a supplement in a *T. reesei* cellulase mixture, Mut M presented more efficient saccharification of sugarcane bagasse than wild-type D2-BGL.

## Methods

### Strains and expression vectors

D2-BGL was expressed in *Pichia pastoris* SMD 1168 (Invitrogen, USA) or in *Saccharomyces cerevisiae* ATCC 4,014,317. Plasmids pGAPZαC with a Zeocin-resistance marker and YEp352 with an URA3 auxotrophic marker (provided by Prof. Frances Arnold, Division of Chemistry, Caltech, Pasadena, CA, USA) were used for recombinant enzyme expression in *P. pastoris* and *S. cerevisiae*, respectively. These two plasmids contain the α-mating factor secretion signal peptide upstream of D2-BGL for protein secretion. The expression vector pGAPZαC-D2 was generated as described in a previous study [20], whereas the expression vector YEp-D2 was generated by cloning the PCR product of D2-BGL into the YEp352 plasmid.

### Heterologous expression of D2-BGL in yeast

*P. pastoris* competent cells were prepared by a modified lithium acetate–dithiothreitol (LiAc–DTT) method [34]. Four hundred nanograms of pGAPZαC-D2, linearized using the restriction enzyme BspHI, was transformed into *P. pastoris* by electroporation. Transformants were selected on YPDS plates (1% yeast extract, 2% peptone, 2% glucose, 1 M sorbitol and 20 g/L agar) supplemented with 100 mg/L Zeocin (Invivogen, Taiwan). For D2-BGL expression in flask culture, one single colony was inoculated in 3 mL of YPD medium (1% yeast extract, 2% peptone, 2% glucose) with Zeocin and then incubated at 30 °C overnight at 250 rpm. This pre-culture was inoculated into 50 mL of YPD medium with the initial OD_600_ value adjusted to 0.05, and then incubated at 30 °C for 3 days at 250 rpm.

*Saccharomyces cerevisiae* competent cells were prepared and transformed using the *S.c.* EasyComp™ Transformation Kit (ThermoFisher Scientific, USA). One hundred nanograms of Yep-D2 was transformed into *S. cerevisiae*, and transformants were then selected on YNB–Ura plates containing 6.7 g/L yeast nitrogen base without amino acids (Sigma-Aldrich, USA), 0.77 g/L –Ura DO Supplement (Clontech, USA), 2% glucose and 20 g/L agar, pH 5.8. For D2-BGL expression, one single colony was inoculated in 4 mL of SDCAA medium containing 6.9 g/L yeast nitrogen base, 5 g/L Bacto casamino acid (BD, USA), 5.4 g/L Na_2_PO_4_, 8.56 g/L NaH_2_PO_4_·H_2_O and 20 μg/mL L-tryptophan (Sigma-Aldrich, USA) [35], and then incubated at 30 °C overnight at 250 rpm. This pre-culture was inoculated into 40 mL of SDCAA medium with the initial OD_600_ value adjusted to 0.1, and then incubated at 30 °C for 2 days at 250 rpm.

### Site-directed mutagenesis

Point mutations were generated in D2-BGL using the QuikChange Lightning Site-Directed Mutagenesis Kit (Agilent, USA). Polymerase chain reaction (PCR) amplification was performed with 20 ng of plasmid as template and back-to-back primers (Additional File 1: TableS2). PCR was initiated with a denaturation step at 95 °C for 2 min, followed by 18 cycles of denaturation at 95 °C for 30 s, annealing at 60 °C for 30 s and extension at 68 °C for 3 min, and the reaction was completed after a final extension step at 68 °C for 5 min. The plasmid template was eliminated by DpnI restriction enzyme digestion at 37 °C for 1 h, and the remaining PCR product was transformed into *E. coli* DH5α strain.

### Random mutagenesis using ePCR

The mutant library was generated and screened in *S. cerevisiae* by following a modified version of a directed evolution approach [36]. The expression vector YEp-D2 was linearized by restriction enzyme digestion to remove the D2-BGL sequence. The mutated D2-BGL sequence was generated using ePCR with 100 ng of YEp-D2 as template in 100 μL PCR solution containing 0.2 mM dNTP, 0.1 μM Sc ePCR F sense primer, 0.1 μM Sc ePCR R antisense primer, 0.5 mM MgCl_2_, 0.2 mM MnCl_2_ and 5 U GoTaq Flexi DNA Polymerase (Promega, USA). The Sc ePCR F and Sc ePCR R primers (Additional File 1: TableS2) generated overlapping regions of 41 base pairs (bp) and 47 bp with linearized yEP vector at the 5′ and 3′ extremities of ePCR products, respectively. ePCR was initiated with a denaturation step at 95 °C for 2 min, followed by 28 cycles of denaturation at 95 °C for 30 s, annealing at 50 °C for 30 s and extension at 72 °C for 3 min, and the reaction was completed after a final extension step at 72 °C for 5 min. Both linearized YEp and ePCR products were purified using the QIAquick Gel Extraction Kit (QIAGEN, Germany).

### Mutant library screening

To construct the D2-BGL mutant library in *S. cerevisiae*, equal amounts (~ 45 fmol) of linearized YEp and ePCR products were transformed together into 40 μL of competent cells, and the transformants were selected on YNB–Ura plates at 30 °C for 3 days. Single colonies were isolated and inoculated individually into the wells of a 96 deep-well plate containing 50 μL of SDCAA medium in each well. Transformants generated with PCR products from wild-type D2-BGL were inoculated into a column of eight wells to serve as a positive control. After a 2-day incubation, 300 μL of SDCAA medium was added to each well and the plate was inoculated at 30 °C for 3 more days. To screen D2-BGL mutants, 50 μL of culture supernatant was mixed with 50 μL of 4 mM *p*-nitrophenyl β-D-glucopyranoside (*p*NPG) in a 96-well plate. Enzyme activity was assayed at 55 °C for 5 min, and the reaction was stopped by adding 100 μL of 1 M Na_2_CO_3_. Values of OD_405_ were measured using a SynergyMx Microplate Reader (BioTek, USA). Average (AVE) and standard deviation (STDEV) were calculated for the positive control (WT) wells of each plate. Transformants with OD_405_ > (AVE_WT_ + 2 × STDEV_WT_) were isolated and cultured in new YNB–URA plates. Plasmids were extracted from potentially improved D2-BGL mutants using a Zymoprep Yeast Plasmid Miniprep II kit (ZYMO RESEARCH, USA). The selected transformants were subjected to flask culture, and the culture supernatants were used for cellobiase activity assay.

### Protein sequence and structure analyses

Multiple alignment of β-glucosidases from *Chaetomella raphigera* D2-BGL (PDB code: 6JXG), *Aspergillus aculeatus* BGL1 (PDB code: 4IIH), *Aspergillus niger* BGL1 (GenBank: RDH24713.1), *Aspergillus niger* ASKU28 (GenBank: AFW98805.1) and *Trichoderma reesei* (PDB: 4I8D) was carried out with Clustal Omega [37]. Structure models of D2-BGL mutants F256Y and F256M were built using SWISS-MODEL [38]. Protein 3D crystal structures were visualized using the PyMOL Molecular Graphics System v2.2.3 (Schrodinger, LLC).

### Enzyme purification by immuno-affinity chromatography

Immuno-affinity chromatography was carried out using a Poly-Prep chromatography gravity-flow column (Biorad, USA) filled with 1 mL CNBr-Activated Sepharose 4B resin (GE Healthcare Bio-sciences AB, Sweden) coupled to purified rabbit polyclonal anti-D2-BGL antibodies (LTK BioLaboratories, Taiwan). To prepare samples, 100 mL *S. cerevisiae* culture supernatant or 30 mL *P. pasto*ris culture supernatant were filtered through 0.2 μm Supor Membrane Disc Filters (PALL, USA). The supernatant was concentrated to 1 mL using a Vivaspin 20 MWCO 30,000 column (GE Healthcare, UK), and buffer exchange was performed three times with 19 mL of binding buffer (20 mM sodium phosphate, pH 7). The final sample was diluted to 10 mL using binding buffer. Purification started with an equilibration step using 10 mL of binding buffer. After sample injection, the column was washed with 30 mL binding buffer. Elution was performed with 15 mL elution buffer (0.1 M glycine, pH 2.7), before adding 700 μL neutralizing buffer (1 M Tris, pH 9). The solution of purified protein was buffer-exchanged and concentrated using a Vivaspin 6 MWCO 10,000 column with 50 mM sodium acetate buffer (NaOAc, pH 5).

### Deglycosylation by Endo H treatment

Purifid D2-BGL and samples from culture supernatant were deglycosylated with endoglycosidase H (NEB, USA) according to the manufacturer’s protocol. Briefly, for the deglycosylation of purified D2-BGL, 2 μg of Pp D2-BGL or Sc D2-BGL were added into 10 μL of reaction solution in Glycoprotein Denaturing Buffer (NEB, USA). After heating at 100 °C for 10 min, the deglycosylation was performed with 0.4 μL Endo H in GlycoBuffer 3 at 37 °C for 1 h. For the deglycosylation of samples used in Western blotting analysis, 10 μL of culture supernatant or serial diluted purified D2-BGL (original concentration: 0.11 mg/mL) were added into the reaction solution (total volume: 50 μL) containing 0.5 μL Endo H in GlycoBuffer 3. The reaction was performed at 37 °C for 30 min.

### Cellulase activity assays

β-Glucosidase activity assays were performed according to the methods described in our previous study [20]. The reaction was assayed at 5, 10 and 20 min to ensure that the measurement is in the linear range. Briefly, β-glucosidase activity was determined with *p*NPG or cellobiose as substrate in 50 mM NaOAc, pH 5. For the *p*NPGase assay, the enzymatic reaction was performed with 100 μL of enzyme solution mixed with 100 μL of 4 mM *p*NPG at 55 °C for 5 min. After adding 600 μL of 1 M Na_2_CO_3_ to stop the reaction, 200 μL of the final solution was transferred into a 96-well plate, and the OD_405_ value was measured using the SynergyMx Microplate Reader. Product concentrations were determined based on a standard curve established by means of *p*NP serial dilution. For the cellobiase assay, 100 μL of enzyme solution was mixed with 100 μL of 20 mM cellobiose, and the reaction was performed at 55 °C for 10 min. The reaction was stopped by heat inactivation at 100 °C for 10 min. Glucose concentrations were determined using a YSI 2700 Select Biochemistry Analyzer (Yellow Springs Instruments, USA). One enzyme unit (U) was defined as 1 μmol of product (i.e., *p*NP or glucose) released per minute.

Total cellulase activity was determined with 50 mg NO.1 filter paper (Whatman, UK). Product concentrations were determined using the dinitrosalicylic acid (DNS) method [39], and a serial dilution of glucose was employed to establish the standard curve of reducing sugar. For the filter paper (FP) assay, 0.5 mL of enzyme solution was mixed with 1 mL of NaOAc buffer (pH 5) containing the filter paper, and the reaction was carried out at 55 °C for 60 min. The reaction was stopped by adding 3 mL DNS solution, and coloration was carried by heating at 100 °C for 5 min. The OD_540_ value was measured using the SynergyMx Microplate Reader. One filter paper unit (FPU) was defined as 1 μmol of reducing sugar released per minute.

### Temperature and pH stability assays

Enzyme solutions containing 1.2 mg/L of purified D2-BGL were incubated at 25–70 °C for 4 h for temperature stability assay, and at pH 4–8 for 24 h for pH stability assays. Relative activity was determined using cellobiase assay.

### Enzyme kinetics

Specific activities were determined using 0.15 μg of purified enzyme in 100 μL enzyme solution (i.e., enzyme concentration: 1.5 mg/L). The *p*NPGase assay was performed at 55 °C for 2 min with 0.25–6 mM *p*NPG, and the cellobiase assay was performed at 55 °C for 5 min with 0.625–100 mM cellobiose. Glucose inhibition was determined via *p*NPG assay in the presence of 0, 5 or 10 mM of glucose. The kinetic parameters *K*_m_, *V*_max_, *K*_i glucose_ and *K*_i substrate_ were determined using the built-in enzyme kinetics models of Prism 8.3.0 (GraphPad Software Inc., USA).

### Intracellular protein extraction

Intracellular proteins were extracted from cell pellets of 3-day culture using YeastBuster Extraction Reagent (Merck, USA). Extraction was performed according to the manufacturer’s instructions. Briefly, 5 μL Extraction Reagent and 0.05 μL 100X Tris (hydroxypropyl) phosphine (THP) solution per mg of cell (wet weight) were used to re-suspend the cell pellet. After adding 25 U of Benzonase Nuclease, the solution was incubated at room temperature for 20 min under agitation. Soluble proteins were collected after concentration at 16,000 g at 4 °C for 20 min, and the protein concentration was determined using the Pierce Detergent Compatible Bradford Assay Kit (ThermoFisher Scientific, USA).

### Western blotting

Protein samples were separated using a 10% SDS-PAGE gel. Western blotting was performed using Amersham Hybond 0.2 μm polyvinylidene difluoride PVDF blotting membrane (GE Healthcare Life Science, Germany) and the Mini Trans-Blot cell system (Bio-Rad, USA). After transfer at 100 V and 200 mA with the PowerPac 1000 Electrophoresis Power Supply (Bio-Rad, USA) for 1.5 h on ice, the PVDF membrane was immersed overnight at 4 °C in a blocking solution containing 30 mL Tris Buffered Saline (TBS; Omics Bio, Taiwan), 1.5 g skim milk powder and 0.05% Tween 20 (J.T.Baker, USA). Anti-D2-BGL serum antibodies were then added at a dilution of 1:30,000 into the blocking solution, and immersion continued at room temperature for a further 1 h. The membrane was washed three times with 30 mL TBS-T (TBS and 0.05% Tween 20), then the membrane was immersed in 10 mL TBS-T containing 0.1 g skim milk powder and anti-rabbit IgG HRP (Perkin Elmer, USA; 1:10,000). After immersion for 1 h, the PVDF membrane was washed three times with TBS-T, and the presence of D2-BGL was revealed by chemiluminescence using Western Lightning ECL Pro (Perkin Elmer, USA). Protein amounts were quantified using the imaging software ImageJ [40].

### Quantitative polymerase chain reactions (qPCR)

Total RNA from *P. pastoris* cells collected after 24-h incubation at 30 °C was isolated using TRIzol Reagent (ThermoFisher Scientific, USA) according to the manufacturer’s instructions. One microgram of DNase I (Invitrogen, USA) treated total RNA was used to generate cDNA using SuperScript II Reverse Transcriptase (Invitrogen, USA). The qPCR reaction solution was prepared with Power SYBR Green PCR Master Mix (Applied Biosystems, USA), and amplification was performed using the QuantStudio™ 12 K Flex system (Applied Biosystems, USA). The *P. pastoris* actin gene (*ACT1*; GenBank: AF216956.1) was used as a reference gene, and the − 2^ΔΔCT^ method was applied to determine relative expression of D2-BGL and unfolded protein response (UPR)-related genes *HAC1*, *KAR2*, *PDI1*, *ERO1* and *CNE1* (with primers listed in Additional File 1: TableS2).

### Biomass saccharification assays

Acid-pretreated biomass was provided by the Institute of Nuclear Energy Research (Taoyuan, Taiwan). The cellulose content in pretreated sugarcane bagasse and rice straws was estimated at 44.7% and 48.17%, respectively. The saccharification assays were carried out with 1, 2, 5 or 7.5% sugarcane bagasse (w/v) in 1 mL reaction solution in a 2 mL Safe-Lock Tube (Eppendorf), The reaction solution was prepared, per 1% of biomass, with 10 μL Tween 80, 0.06 FPU of C1.5L (Sigma-Aldrich, USA) and 0.3 μg of WT D2 or Mut M in 50 mM NaOAc (pH 5). The saccharification was assayed at 50 °C for 72 h and at 250 rpm. Glucose concentration was determined using the glucose analyzer YSI 2700.

## Supplementary Information

**Additional file1: Figure S1. ***S. cerevisiae-*expressed D2-BGL has a higher level of N-glycosylation than that in *P. pastoris*-expressed D2-BGL. *P. pastoris*-expressed D2-BGL (Pp D2-BGL) and *S. cerevisiae*-expressed D2-BGL (Sc D2-BGL) without (-) or with ( +) deglycosylation treatment by Endoglucanase H (Endo H) were used to perform the SDS-PAGE analysis. The theoretical molecular weight of D2-BGL (722 amino acid residues) is 76 kDa. One microgram of purified D2-BGL was loaded per well. **Figure S2.** Substitution of F256 modifies the substrate affinity towards cellobiose in D2-BGL. The substrate binding sub-site + 1 is formed by W34, Y444 and F256 (stick in orange) in D2-BGL (in grey). In D2-BGL F256Y and F256M mutants, the F256 was substituted by tyrosine Y (stick in green) or by methionine M (stick by blue), respectively. TCB: thiocellobiose. **Figure S3.** Kinetics study of *P. pastoris-*expressed D2-BGL with purified WT D2, F256M and Mut M enzymes. (a and b): Determinations of *K*_m_ and *V*_max_ using cellobiose or *p*NPG as substrates. (c and d) Determinations of the inhibition constant of glucose *K*_i glucose_ using *p*NPG as substrate for WT D2 and Mut M. (e) Kinetics model used for substrate inhibition study. Enzyme assays were performed at least in triplicate, and error bars represent the standard deviation. **Table S1.** Mutations generated by error-prone PCR in Mut A, Mut B and Mut C. **Table S2.** Primer list.

## Data Availability

The datasets used and/or analyzed during the current study are available from the corresponding author on reasonable request.

## Abbreviations

*p*NPG: *p*-Nitrophenyl β-D-glucopyranoside
DTT: Dithiothreitol
DNS: Dinitrosalicylic acid
FP: Filter paper
PVDF: Polyvinylidene difluoride
