# Supplementary material for: Improvements of the productivity and saccharification efficiency of the cellulolytic β-glucosidase D2-BGL in Pichia pastoris via directed evolution
Source: Biotechnol Biofuels. 2021 May 31;14:126. doi: 10.1186/s13068-021-01973-3 (PMC8166090; doi:10.1186/s13068-021-01973-3)
Supplement: Supplementary file 1 — Additional file1: Figure S1. S. cerevisiae-expressed D2-BGL has a higher level of N-glycosylation than that in P. pastoris-expressed D2-BGL. P. pastoris-expressed D2-BGL (Pp D2-BGL) and S. cerevisiae-expressed D2-BGL (Sc D2-BGL) without (-) or with ( +) deglycosylation treatment by Endoglucanase H (Endo H) were used to perform the SDS-PAGE analysis. The theoretical molecular weight of D2-BGL (722 amino acid residues) is 76 kDa. One microgram of purified D2-BGL was loaded per well. Figure S2. Substitution of F256 modifies the substrate affinity towards cellobiose in D2-BGL. The substrate binding sub-site + 1 is formed by W34, Y444 and F256 (stick in orange) in D2-BGL (in grey). In D2-BGL F256Y and F256M mutants, the F256 was substituted by tyrosine Y (stick in green) or by methionine M (stick by blue), respectively. TCB: thiocellobiose. Figure S3. Kinetics study of P. pastoris-expressed D2-BGL with purified WT D2, F256M and Mut M enzymes. (a and b): Determinations of Km and Vmax using cellobiose or pNPG as substrates. (c and d) Determinations of the inhibition constant of glucose Ki glucose using pNPG as substrate for WT D2 and Mut M. (e) Kinetics model used for substrate inhibition study. Enzyme assays were performed at least in triplicate, and error bars represent the standard deviation. Table S1. Mutations generated by error-prone PCR in Mut A, Mut B and Mut C. Table S2. Primer list. [file 13068_2021_1973_MOESM1_ESM.pptx]

## Slide 1
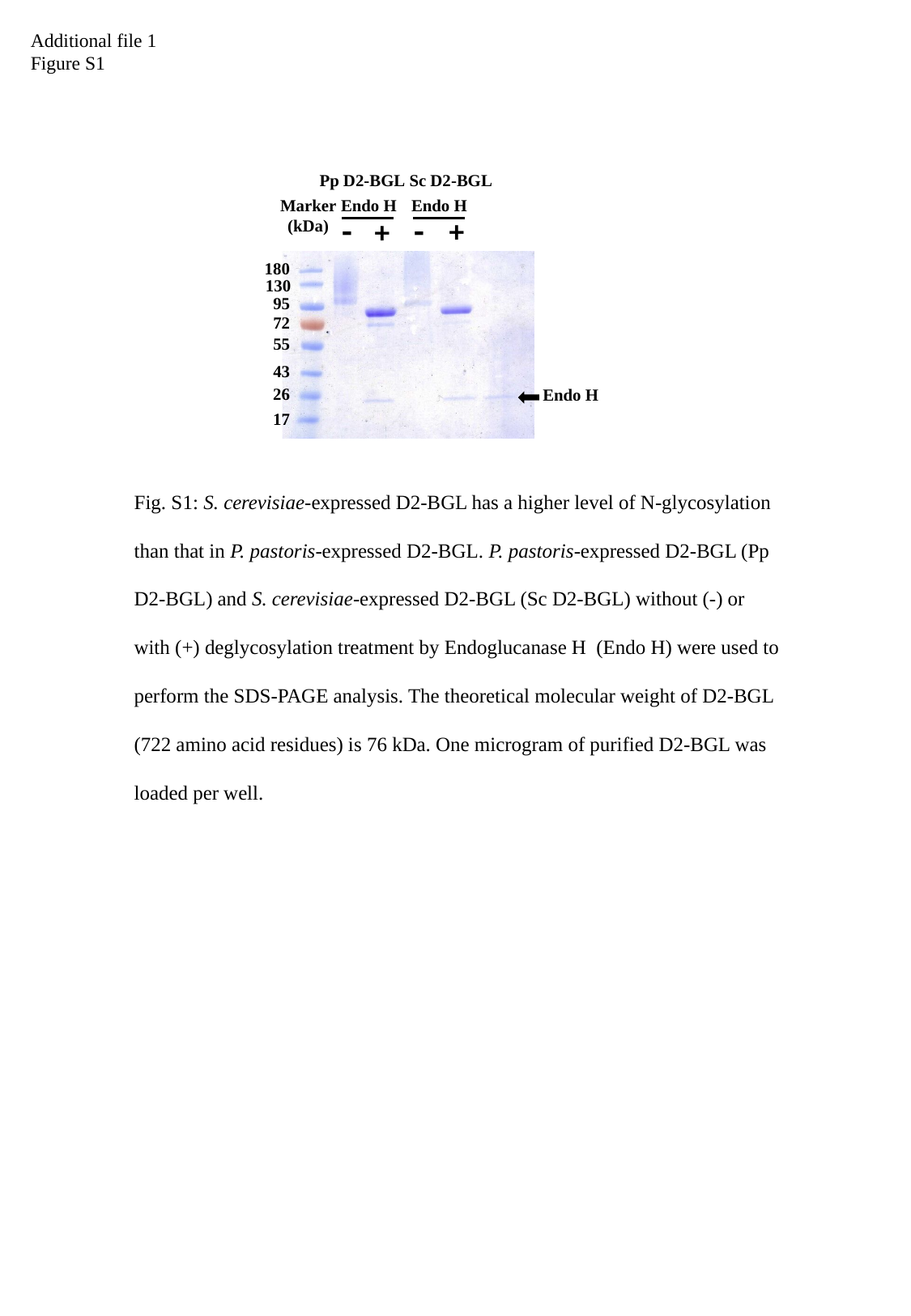

Additional file 1
Figure S1
Pp D2-BGL
Sc D2-BGL
Marker
(kDa)
Endo H
Endo H
-
+
180
130
95
72
55
43
26
Endo H
17
-
+
Fig. S1: S. cerevisiae-expressed D2-BGL has a higher level of N-glycosylation than that in P. pastoris-expressed D2-BGL. P. pastoris-expressed D2-BGL (Pp D2-BGL) and S. cerevisiae-expressed D2-BGL (Sc D2-BGL) without (-) or with (+) deglycosylation treatment by Endoglucanase H (Endo H) were used to perform the SDS-PAGE analysis. The theoretical molecular weight of D2-BGL (722 amino acid residues) is 76 kDa. One microgram of purified D2-BGL was loaded per well.

## Slide 2
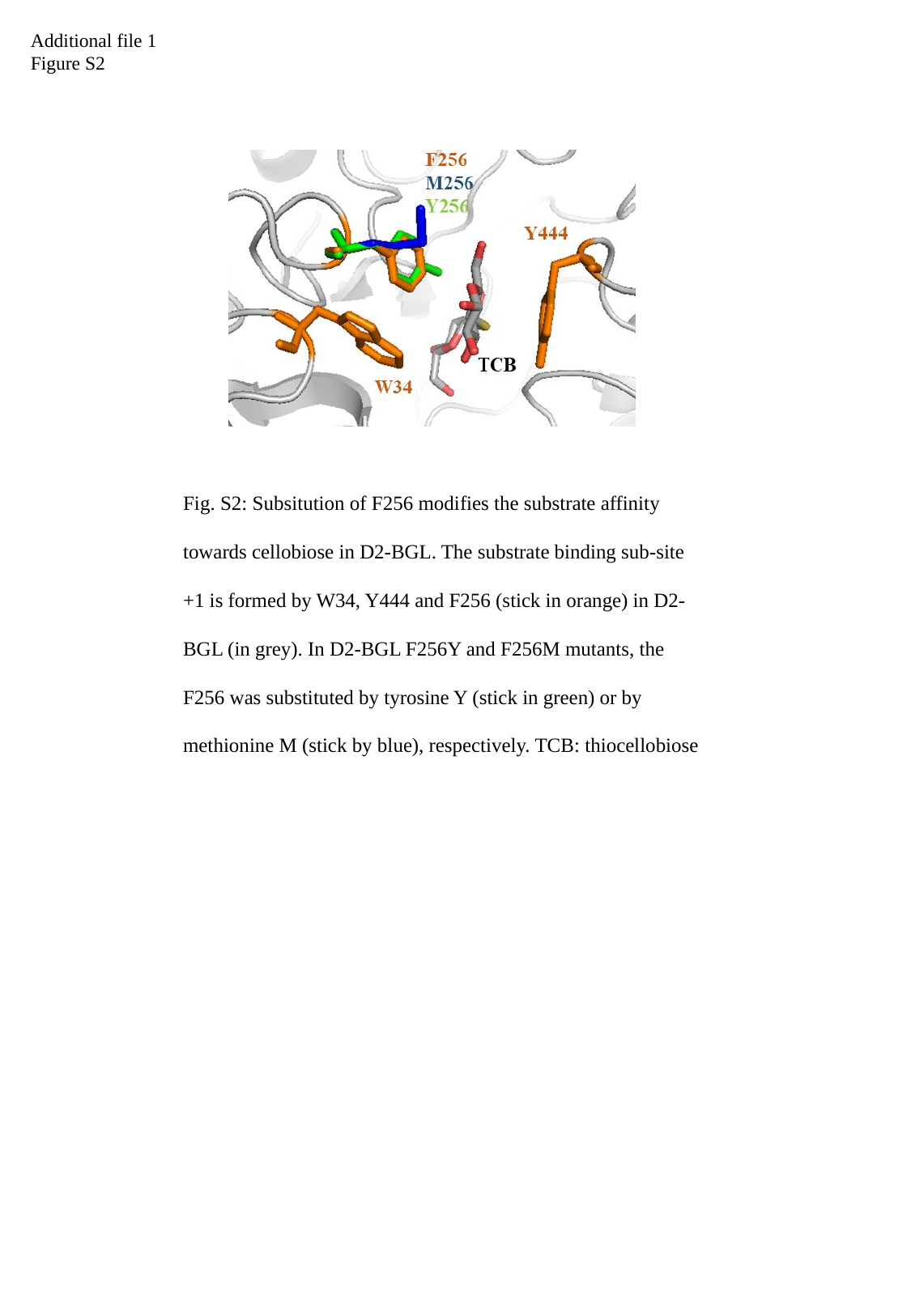

Additional file 1
Figure S2
Fig. S2: Subsitution of F256 modifies the substrate affinity towards cellobiose in D2-BGL. The substrate binding sub-site +1 is formed by W34, Y444 and F256 (stick in orange) in D2-BGL (in grey). In D2-BGL F256Y and F256M mutants, the F256 was substituted by tyrosine Y (stick in green) or by methionine M (stick by blue), respectively. TCB: thiocellobiose

## Slide 3
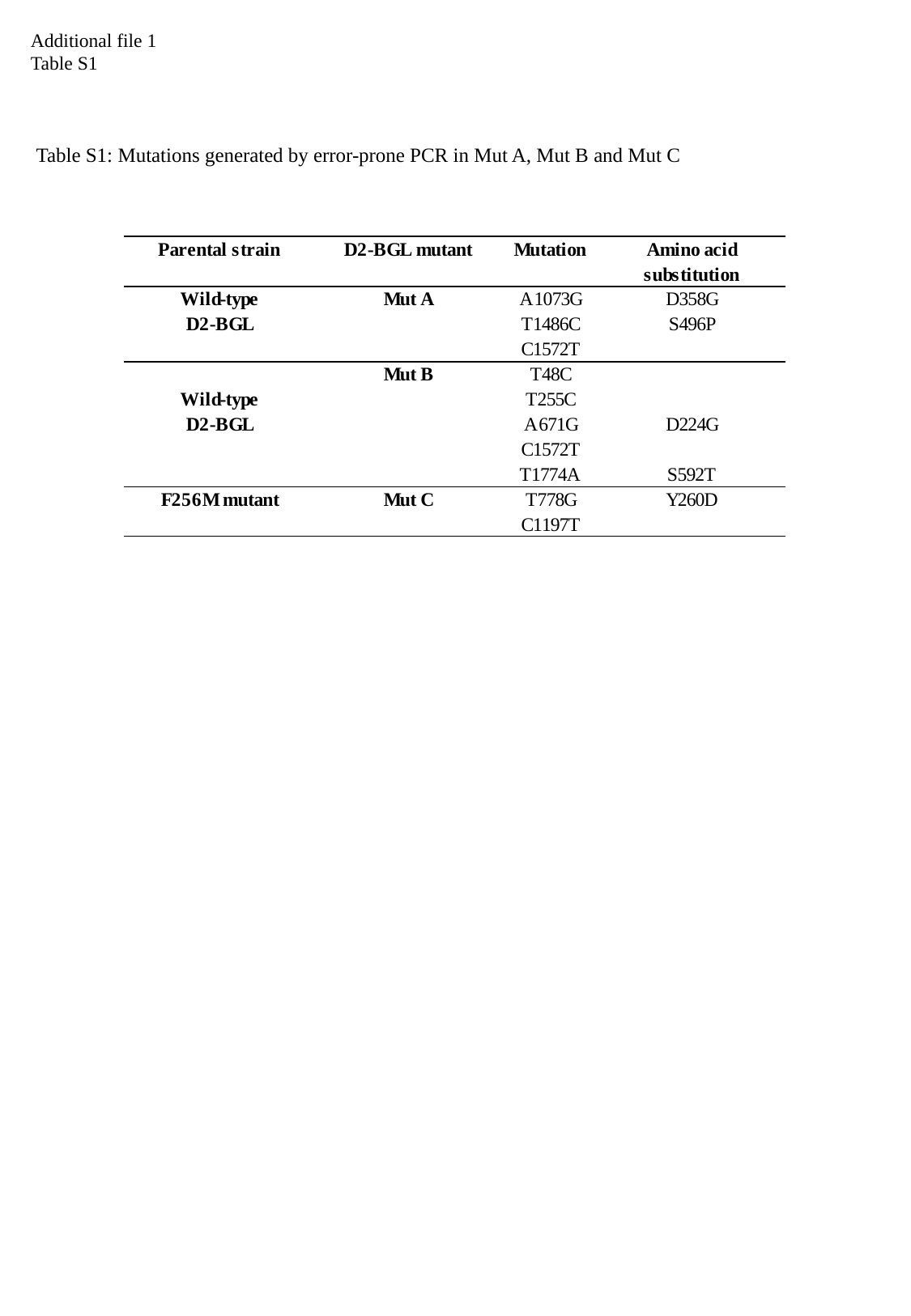

Additional file 1
Table S1
Table S1: Mutations generated by error-prone PCR in Mut A, Mut B and Mut C

## Slide 4
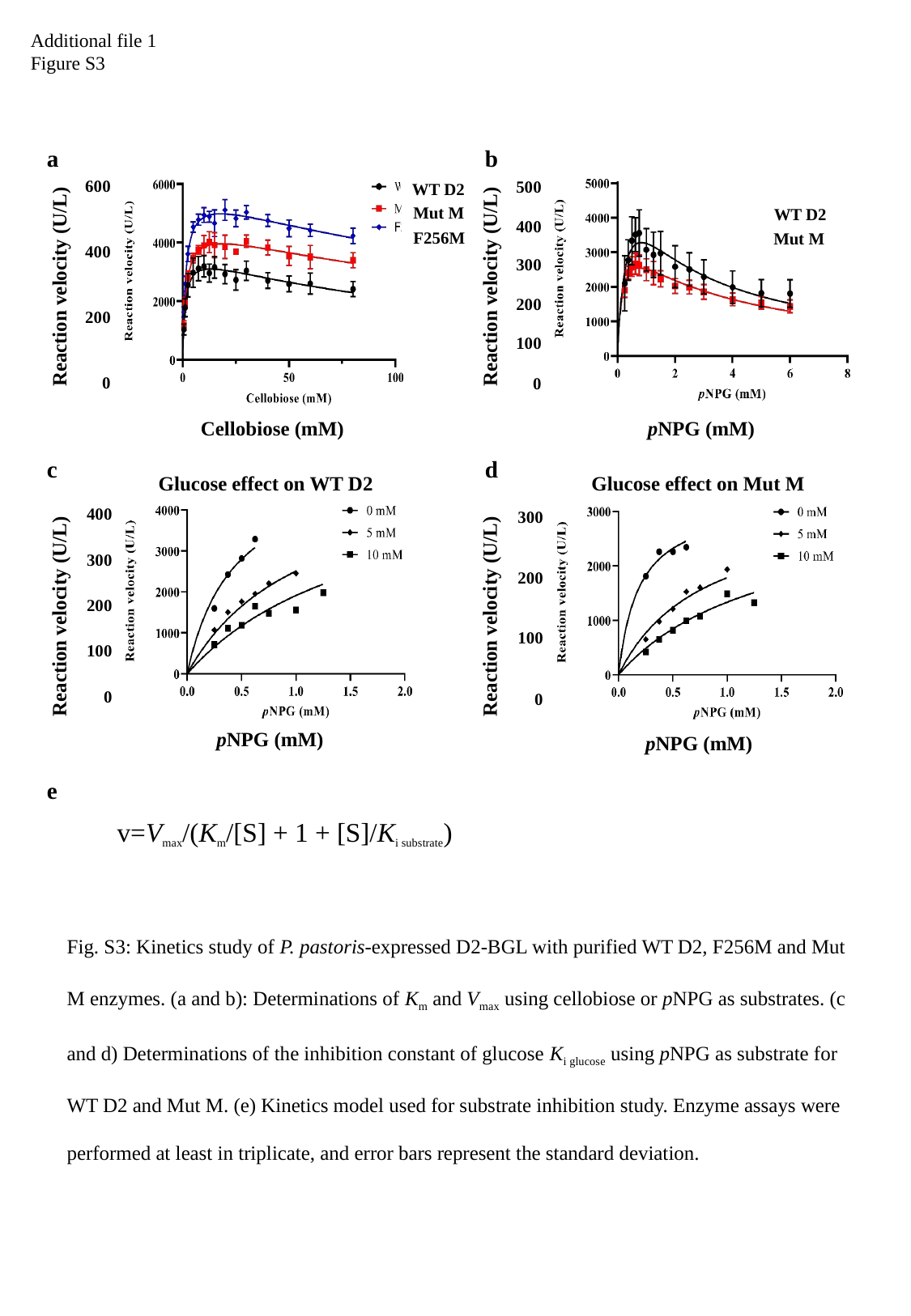

Additional file 1
Figure S3
a
b
600
500
WT D2
Mut M
WT D2
400
F256M
Mut M
400
300
Reaction velocity (U/L)
Reaction velocity (U/L)
200
200
100
0
0
pNPG (mM)
Cellobiose (mM)
c
d
Glucose effect on WT D2
Glucose effect on Mut M
400
300
300
200
200
Reaction velocity (U/L)
Reaction velocity (U/L)
100
100
0
0
pNPG (mM)
pNPG (mM)
e
v=Vmax/(Km/[S] + 1 + [S]/Ki substrate)
Fig. S3: Kinetics study of P. pastoris-expressed D2-BGL with purified WT D2, F256M and Mut M enzymes. (a and b): Determinations of Km and Vmax using cellobiose or pNPG as substrates. (c and d) Determinations of the inhibition constant of glucose Ki glucose using pNPG as substrate for WT D2 and Mut M. (e) Kinetics model used for substrate inhibition study. Enzyme assays were performed at least in triplicate, and error bars represent the standard deviation.

## Slide 5
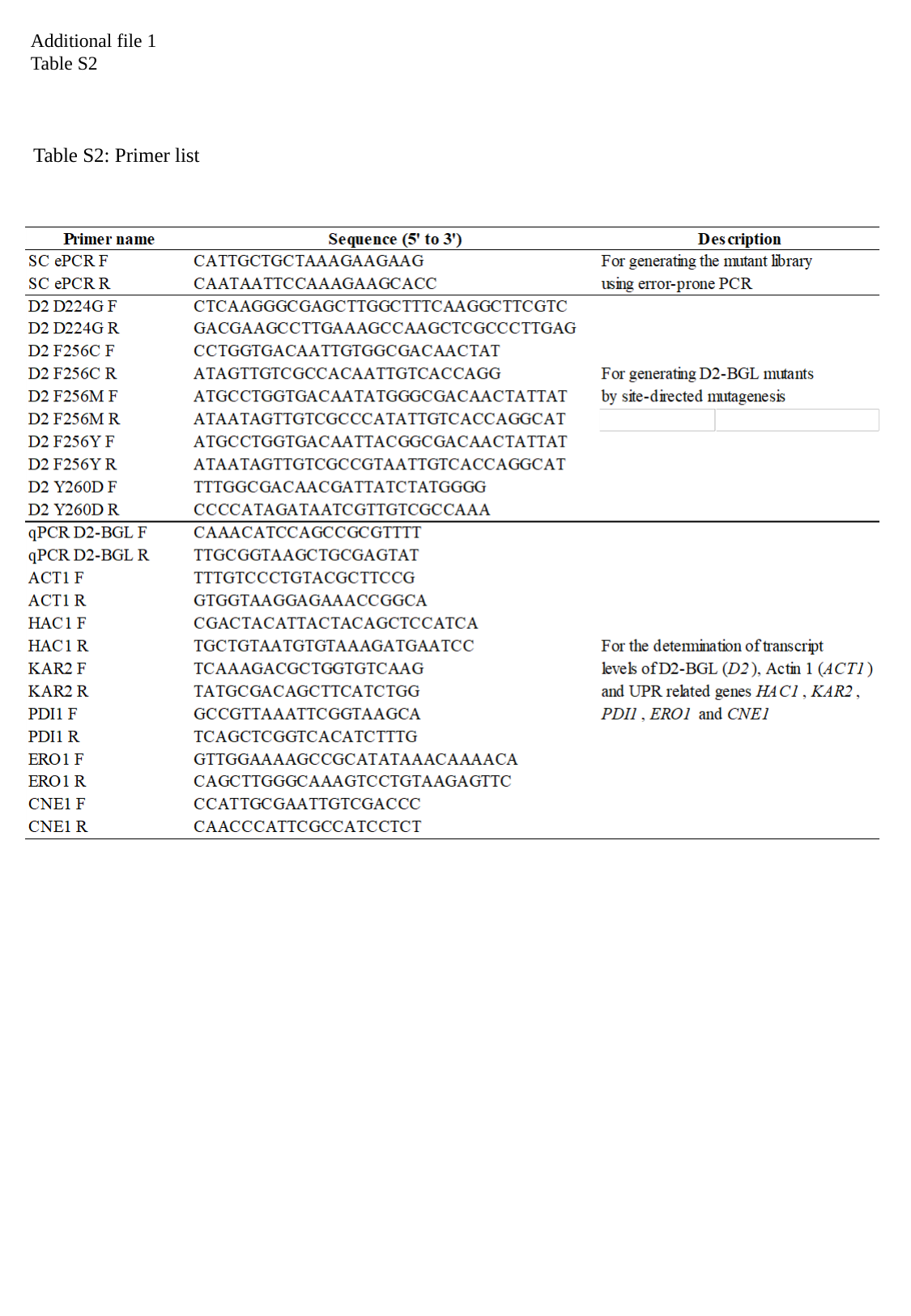

Additional file 1
Table S2
Table S2: Primer list
